# Formulation and Evaluation of Taste Masked Mouth Dissolving Tablets of Levocetirizine Hydrochloride

**Published:** 2012

**Authors:** Vijay Sharma, Himansu Chopra

**Affiliations:** a *Faculty of Pharmacy, P.R.C., PRIST University, Thanjavur (T.N.), India. *; b *Faculty of Pharmacy, GRD (PG) IMT, 214-Rajpur Road, Dehradun (U.K.), India.*

**Keywords:** Levocetirizine, Tulsion-335, Drug-resin complex, Superdisintegrant

## Abstract

Aim of this research work was to develop mouth dissolving tablet that disintegrates rapidly in mouth by using tasteless complex of Levocetirizine and Tulsion-335. Effect of different parameters such as swelling time, resin activation, drug resin ratio as well as stirring time was optimized by taste and percentage drug loading. Formulated DRC (Drug Resin Complex) was characterized by infrared spectroscopy, thermal analysis and X-ray diffraction pattern. Tablets were formulated by wet granulation with PVP as binder, Sodium Starch Glycolate (SSG) and Crospovidone as super disintegrants. In these batches optimum hardness was achieved but disintegration time was found to be very high as ≥ 70 second, so further trials were planned by using different superdisintegrants such as Croscarmellose sodium, Sodium Starch Glycolate (SSG) as well as Crospovidone by wet granulation method. Tablets formulated with 7.5% crospovidone showed comparatively low disintegration time (25 sec), wetting time (20 sec) and friability (0.60 %) than the other batches. In present study we optimized the conditions required for maximum drug loading of Levocetirizine with Tulsion-335. Among different superdisintergants, crospovidone was found suitable with drug-resin complex to get the low disintegration time, wetting time and friability of tablets.

## Introduction

Among all route of administration, oral route is most important and preferable route of administration for solid dosage forms (1). Tablets are the most common solid dosage form, administered orally, but many patients specially children, mentally ill patients and geriatrics have problem in swallowing the tablets (2, 3). Mouth dissolving tablets (MDT) have advantage of ease of administration and rapid onset of action. Further there is advantage of rapid disintegration without use of water in oral cavity. When MDT is kept in oral cavity then saliva quickly penetrates into tablet pores and causes rapid disintegration (4). To mask the bitterness of drug various techniques are available, among those taste masking by use of ion exchange resin is most commonly used commercially. Ion exchange resins (IERs) are used to mask bitter taste of drug. They are solid and insoluble high molecular weight poly electrolytes capable of exchange of their ions with the counter ion in the surrounding medium (5). A number of superdisintegrants such as croscarmellose sodium, crospovidone and sodium starch glycolate are used for rapid disintegration of tablet (6-13). In present study an attempt was made to mask the taste of Levocetirizine with formulation of mouth dissolving tablet having desired good characteristics of MDT so as to give pleasant taste and good bioavailability.

## Experimental

Levocetirizine hydrochloride was obtained from Yegna Manojavam Drug and Chemicals ltd. (A.P.). Croscarmellose Sodium was obtained from DMV International, Crospovidone from FMC Biopolymer and Sodium Starch Glycolate from Rama production company. Polacrilin Potassium was purchased from Corel Pharma Pvt. Ltd. and MCC from Gujarat Microwax Pvt. Ltd.

*Formulation of drug resin complex*

Formulation of DRC was done by the batch process; 100 mg of resin Tulsion-335 was placed in a beaker containing 25 mL of deionized water and allowed to swell for a definite period of time. Accurately weighed amount of Levocetirizine hydrochloride (as per 1 : 3 and 1 : 5 drug resin ratio) was added and stirred for desired period of time. The mixture was filtered and residue was washed with deionized water. Filtrate was analyzed by U.V. spectrophotometer at 231 nm for the unbound drug and percentage drug loading was calculated.

*Optimization of drug resin ratio and stirring time*

Separate batches of drug-resin complex were prepared by altering the ratio of drug and resin as 1 : 3 and 1 : 5. Firstly the resin was soaked into 25 mL of deionized water contained in a beaker for 30 min and then the drug was added and stirred for different time period as 30, 60, 120 and 240 min. The complexation in batch process was performed and percentage drug loading and taste were determined.

*Optimization of resin activation*

Accurately weighed resin (25 mg), was placed on a filter paper in a funnel and then it was washed with double distilled water and subsequently with 1 N HCl (100 mL) for acid activation. The resin was rewashed with water until neutral pH was reached. Similarly, alkali activation of resin was performed, by replacing 1 N HCl with 1N KOH. For Acid- alkali activation, resin was treated with 1 N HCl and 1N KOH (1 N HCl: 1N KOH = 50:50). This activated resin was used for complexation process. The drug resin ratio, swelling time and stirring time kept constant for the DRC formulation by batch process and percentage drug loading was determined. 

*Optimization of swelling and stirring time*

Optimization of swelling and stirring time was done by changing swelling time as well as stirring time. For different batches Tulsion-335 (200 mg) was soaked in 250 mL of deionized water in a beaker for 40, 60, 90 and 120 min respectively. The complexation by batch process adopted for the formation of DRC by stirring for 240 and 300 min for different batches and percentage drug loading and taste were determined.

*Optimization of temperature and pH on complex formation*

The complexation of 50 mg of drug with 300mg of resin, slurred in 25 mL of deionized water in a beaker, was performed at 25^0^C, 40^0^C, 60^0^C and 80^0^C using temperature controlled magnetic stirring for 30 min. The volume of filtrate was made up to 50 mL with aqueous washing of DRC. The amount of bound drug was estimated spectrometrically (231 nm) from the unbound drug in filtrate. 

Accurately weighed 50 mg of drug powder was added to 300 mg of resin slurred in 25 mL of different pH (2, 3, 4, 5, 6 and 8) solution prepared from standard solution of HCl and NaOH in a 100 mL beaker and maintained at 25^0^C. The drug loading efficiency was estimated.

*Characterization of DRC*

FTIR spectra was obtained by Jasco FTIR 6100 type A, Japan spectrometer, sample was prepared in KBr disks, and spectra was recorded over the wave number 4000- 400 cm^-1^. All three spectra were completely analyzed. 

The powder X-Ray diffraction pattern of Levocetirizine, Tulsion 335 and DRC were taken by Philips PW 1729 X–ray differectometer Legroup interconnexion, Scient Jurie, Cadlada. Radiation generated from Copper source with wavelength of 30mA at 40 kV and the range of 5x10^-3^ cycles / sec was used. 

A mettler Toledo differential scanning colorimeter 821 (mettle Toledo, Griefensci, Switzerland) equipped with an in cooler and refrigerated cooling system was used to analyze the thermal behavior of Levocetirizine and DRC. Sample (5-10 mg) was heated in hermetically sealed aluminum pans at temperature 20^o^C/min. nitrogen were purged at 50 mL/min and 100 mL/min through cooling unit.

**Table 1:** Formulation of tablet with different superdisintegrants

**Ingredients**	**Batches**											
	**B10**	**B11**	**B12**	**B13**	**B14**	**B15**	**B16**	**B17**	**B18**	**B19**	**B20**	**B21**
Drug:: Tulsion 335 complex	35	35	35	35	35	35	35	35	35	35	35	35
MCC( Avicel pH 101)	77	73.25	69.5	73.25	69.5	77	77	73.25	69.5	77	73.25	69.5
SSG	7.5	11.25	15	**-**	**-**	**-**	**-**	**-**	**-**	-	**-**	**-**
Cross-povidone	**-**	**-**	**-**	-	-	-	-	-	-	7.5	11.25	15
Cross-carmellose	**-**	**-**	**-**	7.5	11.25	15	-	-	-	**-**	-	-
Kyron T-314	**-**	**-**	**-**	-	-	-	7.5	11.25	15	**-**	-	-
Mannitol	20	20	20	20	20	20	20	20	20	20	20	20
Aspartame	6	6	6	6	6	6	6	6	6	6	6	6
Talc	3	3	3	3	3	3	3	3	3	3	3	3
Mag. Stearate	1.5	1.5	15	1.5	1.5	1.5	1.5	1.5	1.5	1.5	1.5	1.5

*Taste characterization*

Taste evaluation was done by a panel of six volunteer using time intensity method. One tablet was in mouth for 10 sec, bitterness level was recorded, written consent was prepared volunteer as per protocol previously prepared.

*Formulation of MDTs*

First of all formulation of MDTs was done by direct compression technique for batch B_1_ to B_3_ by taking DRC equivalent to 5 mg of Levocetirizine HCl. MCC was used as diluent, PVP K 30 as dry binder, mannitol as soothing agent, talc as an antiadherent and magnesium stearate as a lubricant. All the ingredients were accurately weighed and passed through 100 # sieve and mixed with complex. The above powder blend was compressed using rotary tablet machine using 13 mm concave punches. 

Next trial was performed with wet granulation technique. In this process batches B_4_ to B_9_ was formulated with PVP K-30 while batches B_10_ to B_21_ were formulated with different superdisintegrants having different concentrations. All the ingredients were passed through 100# sieve. Binder solution was added to the powder blend. Wet mass was then passed through 18 # sieve and dried at 60.C for 30 min. the dried granules were passed through 20 # and retained on 40 # sieve. Talc and Magnesium stearate were added extra granularly to the granules (before compression process). Then, the granules were compressed using rotary tablet machine.

**Table 2:** Optimization of stirring time and drug resin ratio

**Batch**	**Drug: Resin ratio**	**Swelling Time (Min)**	**Stirring Time (Min)**	**Taste**	**%Drug Loading**
F1	1:3	30	30	+	30.15%
F2	1:3	30	60	+	44.05%

+ Slight taste masking, **++ **taste masked with bitter after taste, **+++ **complete taste masking

**Table 3:** Optimization of stirring time and drug resin ratio

**Batch**	**Drug: Resin ** **ratio**	**Swelling Time ** **(Min)**	**Stirring Time ** **(Min)**	**Taste**	**%Drug Loading**
F3	1:5	30	30	++	49.97%
F4	1:5	30	60	++	50.41%
F5	1:5	30	120	++	52.17%
F6	1:5	30	240	++	70.32%

# All batches contained 5 mg of Levocetirizine HCl

*Characterization of formulated MDTs*

Characterization was done by different parameters such as weight variation, hardness, friability, thickness, *in-vitro* disintegration time, wetting time, *in-vitro* dispersion time and by in-vitro dissolution study. 

*In-vitro release study from drug resin complex and MDT*

Drug release study from drug resin complex was carried out at pH 6.8 using USP dissolution apparatus type II. Weighed quantity of drug-resin complex equivalent to normal dose was suspended in dissolution media and quantity of drug release was determined periodically. 

Drug release study from formulated MDTs was carried out in 900 mL of HCl buffer pH 1.2 using USP dissolution apparatus type II. Rotation speed of paddles was fixed at 50 rpm. Sample is taken from the medium and drug content was determined by taking absorbance in Shimadzu U.V. spectrophotometer (UV-1601).

## Result and Discussion

Optimization of swelling and drug: resin ratio was done by taking inactivated resin in batches F_1_ to F_6_. As in batches F_1_ and F_2_ drug resin ratio kept constant at 1:3 and stirring time varied from 30 min to 60 min in batch F_1_ to batch F_2_, taste was found to be slightly masked but percentage drug loading increases from 30.15% ± 1.04% for 60 min. But in batches F_3_ to F_6_ drug : resin ratio increases to 1:5, swelling time remain constant at 30 min and stirring time varied for 30, 60, 120 and 240 min respectively and percentage drug loading was found to be 49.97% ± 1.16%, 50.41 ± 1%, 52.17% ± 0.41%and 70.32% ± 1.2% wt/wt. respectively and taste was masked. Highest taste masking and percentage drug loading was achieved with drug resin ratio 1:5 and when stirring time was maximum upto 240 min.

**Table 4:** Effect of resin activation

**Batch **	**Resin Activation**	**Drug: Resin ratio**	**Swelling Time** **(Min)**	**Stirring Time (Min)**	**Taste**	**%Drug Loading**
F7	Acid Activation	1:5	30	240	**++**	61.4%
F8	Alkali Activation	1:5	30	240	**++**	45.80%
F9	Acid-Alkali Activation	1:5	30	240	**++**	28.69%

**++ **Taste masked with bitter after taste

**Table 5:** Optimization of effect pH on % drug loading

**pH**	**% drug loading**
2	85.10
3	86.81
4	87.32
5	97.69
6	96.62
7	92.47
8	89.54

Drug: resin ratio 1:5.

The percentage drug loading was also determined with acid treated resin, alkali treated and resin treated with both acid and alkali was found to be 61.40% ± 1.32%, 45.80% ± 0.32% and 28.69% ± 0.62%. wt/wt respectively. Highest percentage drug loading was found for acid activated resin but as compared to inactivated resin no major effect was found on percentage drug loading. Thus further trials were made with inactivated resin.

Complexation process in between drug and resin depends on pKa value of both, involves exchange of ions. Results indicate increase in pH from 2 to 5 also increases percentage drug loading. A maximum drug loading 97.69% wt/wt was found at pH 5. As pH increases above 5, the percentage drug loading decreases. This was due to that pH of solution affects both solubility and degree of ionization of drug and resin. Drug loading is low at lower pH due to -COO^- ^groups of resin and compete with drug for binding.

**Figure 1:**
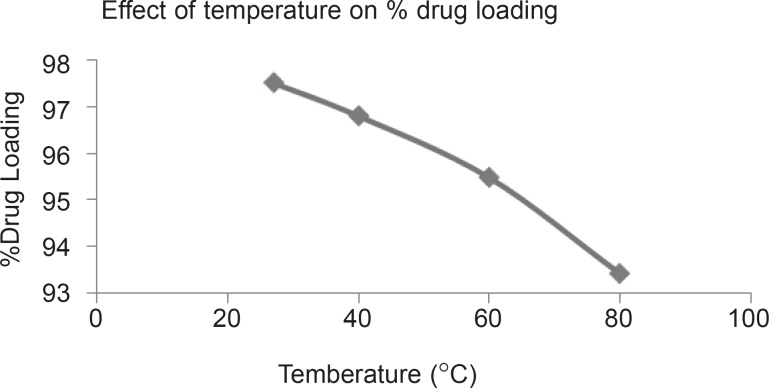
Effect of temperature on % drug loading

Percentage drug loading was determined at temperature 27°C, 40°C, 60°C and 80°C. Result indicate 97.52%, 96.81%, 95.49% and 93.43% drug loading at 27, 40, 60 and 80°C respectively.

Effect of swelling time and stirring time was optimized in batches F_10_ to F_14_, here drug: resin ratio kept constant for 1:5, swelling time and stirring time was changed from batch F_10_ to F_14_. In batch F_11_, complete taste masking was achieved as well as percentage drug loading was found to be 97.33% when swelling time kept as 60 min and stirring time kept 240 min.

**Table 6:** Optimization of swelling and stirring time

**Batch**	**Drug: Resin ** **ratio**	**Swelling Time ** **(Min)**	**Stirring Time ** **(Min)**	**Taste**	**%Drug Loading**
F10	1:5	40	240	+++	70.33%
F11	1:5	60	240	+++	97.33%
F12	1:5	60	300	+++	48.28%
F13	1:5	90	240	+++	70.70%
F14	1:5	120	240	+++	65.01%

+++ Complete taste masking

**Figure 2:**
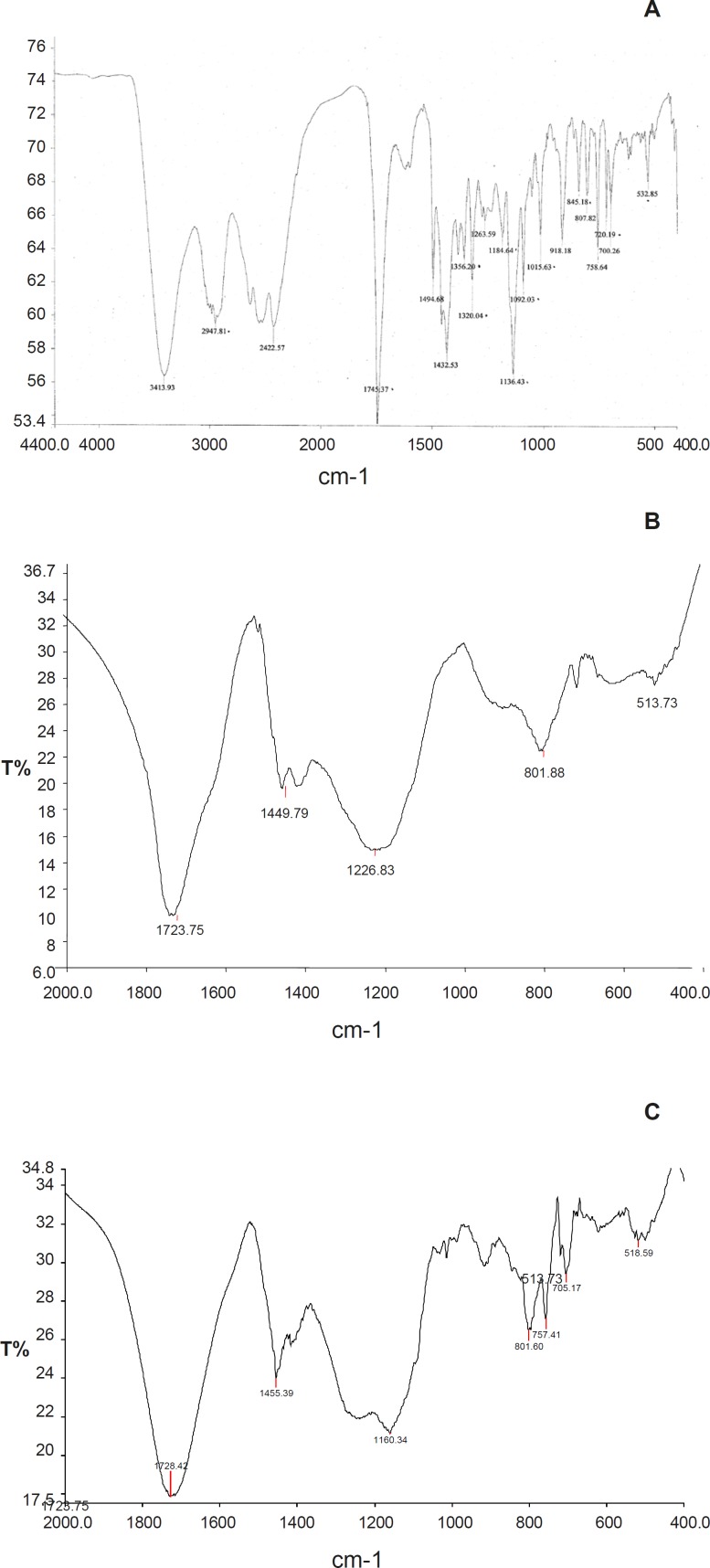
A: Infra red spectra of Levocetirizine HCl. B: Infra red spectra of Tulsion 335. C: Drug Resin Complex IR Spectra

**Figure 3:**
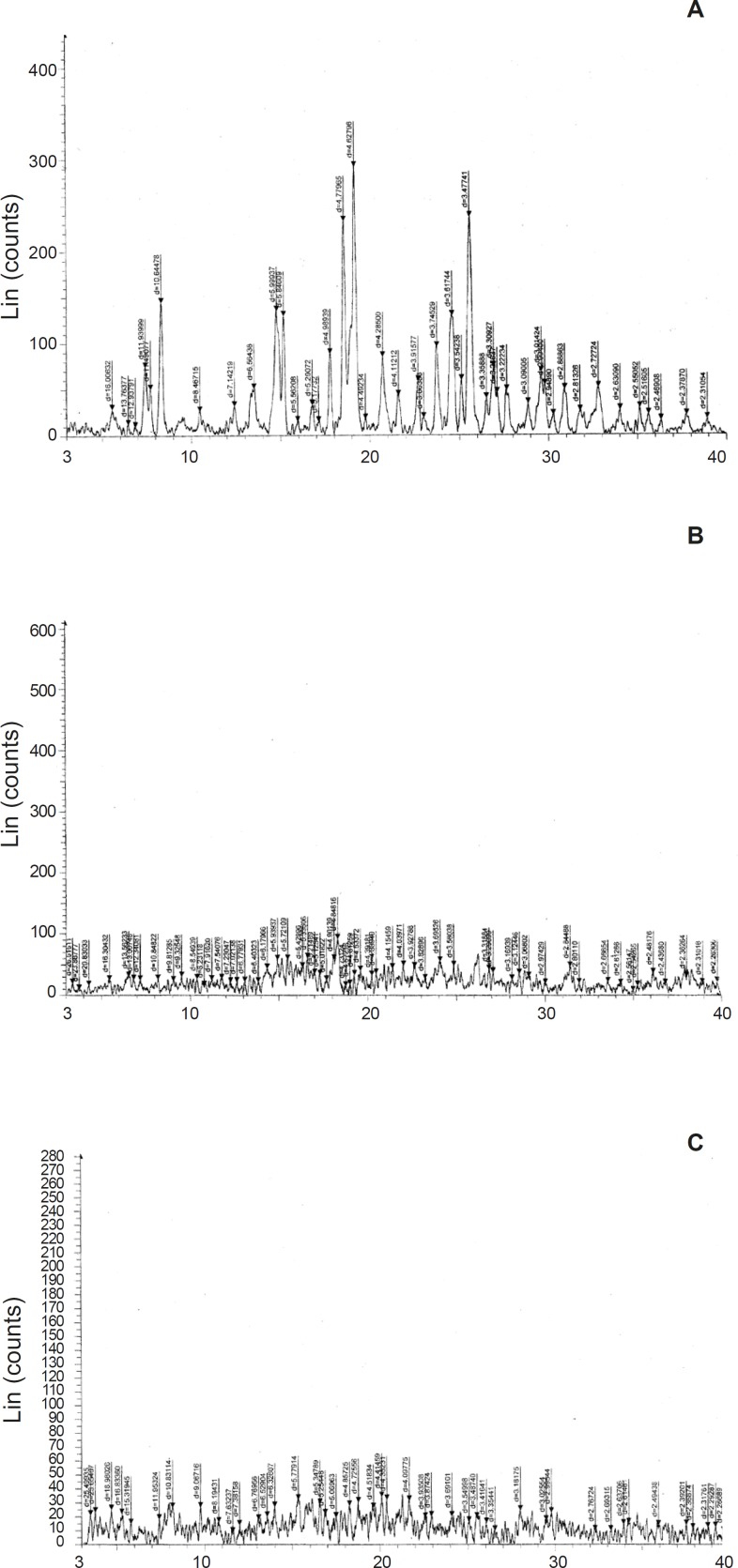
A: XRD Curve of Drug (Levocetirizine HCl). B: XRD Curve of Resin (Tulsion-335). C: XRD Curve of Drug resin complex

Infrared spectra of Tulsion-335, Levocetirizine and DRC were shown in Figure 2. Levocetirizine hydrochloride is crystalline while Tulsion 335 is amorphous in nature. X ray pattern diffraction (XRPD) of Tulsion-335, Levocetirizine and DRC are shown in Figure 3.

Several sharp peaks in XRD spectra of pure drug represent the crystalline nature of drug while a diffused peak in XRD spectra of Tulsion-335 represents amorphous nature of resin. But on the other hand XRD pattern of DRC shows disappearance of characteristic peaks of drug and also found to be broadened, these finding suggest the formation of drug resin complex.

By DSC curves, the thermal behavior of Pure Drug Levocetirizine HCl shows peak endotherm at 213.55°C corresponding to loss of water of crystallization and melting of pure drug. Thermal behavior of Resin (Tulsion 335) shows peak endotherm at 152.28°C while thermal behavior of Drug Resin Complex shows peak endotherm at 70.29°C the reduction of height and sharpness of endotherm is due to loading of drug in resin. That shown, there was no interaction was observed between drug and complex. It concludes that, resin was not affecting the characteristic of drug due to complexation process and indicates the amorphous nature of DRC.

**Figure 4:**
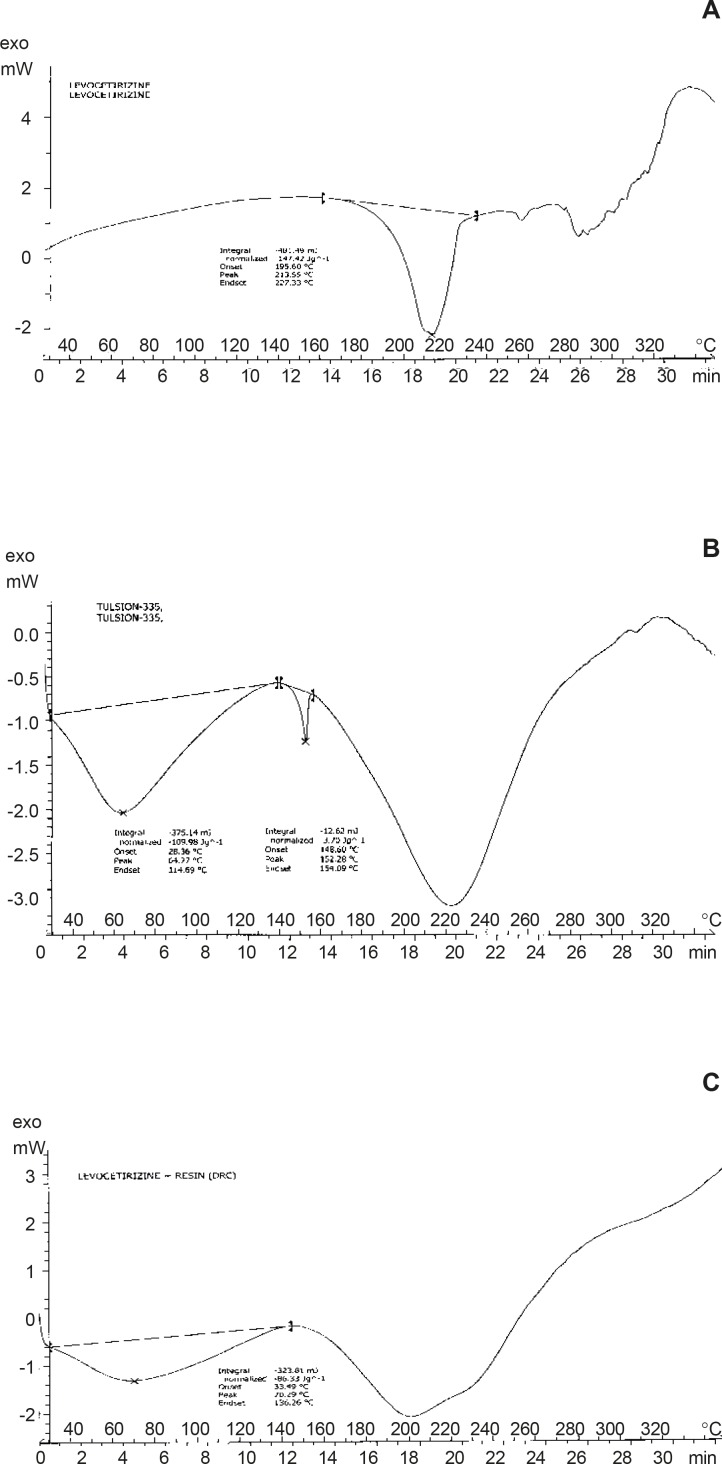
A: DSC curve of drug (Levocetirizine HCl). B: DSC curve of resin (Tulsion-335) C: DSC curve of drug resin complex

**Figure 5:**
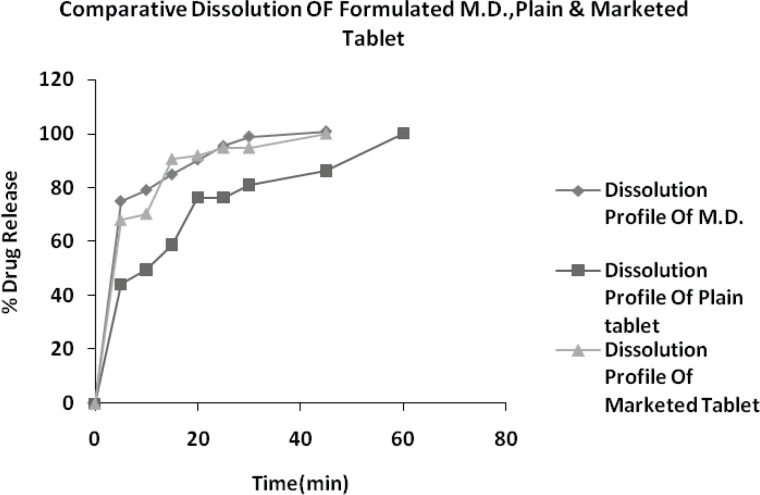
Dissolution profile of optimized formulation, plain tablet and marketed tablet

*In-vitro drug release study*

In-vitro release of drug from drug-resin complex at pH 6.8 was found to be less than 4.8% within 1 minute, indicating the presence of exchangeable ions of ionizable electrolytes in dissolution fluid of pH 6.8. Hence DRC is stable at salivary pH and poor released amount of drug from DRC do not impart bitter taste at the time of administration and while pass to GI tract from mouth. Further drug release profile of optimized formulation (B-20), plain tablet (do not have crospovidone) and marketed brand (Vozet 5 mg tablet) were analyzed in HCl buffer pH 1.2.

## Conclusions

In present study we optimized the conditions required for maximum drug loading of Levocetirizine with Tulsion-335. All the optimized tablet (MDT) formulations of Levocetirizine (B_10_ to B_21_) showed all parameters within limit as well as good physicochemical properties. Drug release rate of formulated MDTs was also found to higher as compared to conventional tablet. Crospovidone 7.5% Batch B_20_ having hardness (3.0 kg/cm^2^), friability (0.60%), wetting time (20 sec) and assay 99.37%, hence tablets formulated with crospovidone not only increases rate of dispersion but also increases rate of drug release.
